# Common risk indicators for oral diseases and obesity in 12-year-olds: a South Pacific cross sectional study

**DOI:** 10.1186/s12889-017-4996-y

**Published:** 2018-01-08

**Authors:** Stéphanie Tubert-Jeannin, Hélène Pichot, Bernard Rouchon, Bruno Pereira, Martine Hennequin

**Affiliations:** 1 University Clermont Auvergne, EA 4847, Centre de Recherche en Odontologie Clinique, BP 10448, 63000 Clermont-Ferrand, France; 2 Sanitary and Social Agency of New Caledonia, Nouméa, New Caledonia; 3 CHU Clermont-Ferrand, Biostatistics Unit, DRCI, 63000 Clermont-Ferrand, France; 4 CHU Clermont-Ferrand, Dental Unit, Clermont-Ferrand, France

**Keywords:** Children, Oral health, Mastication, Obesity, BMI, Waist-to-height ratio, Risk factors, Epidemiology

## Abstract

**Background:**

Despite the increasing need to prevent obesity and oral diseases in adolescents worldwide, few studies have investigated the link existing between these conditions and their common risk factors. This study aims to evaluate the oral health and weight status of New Caledonian Children (aged 6,9,12 years) and to identify, amongst 12-year-olds, risk indicators that may characterize the groups of children affected by oral diseases, obesity or both diseases.

**Methods:**

This survey evaluated in 2011–2012 the oral health and stature-weight status and related risk indicators in a national representative sample of 6, 9 and 12 years-old children in New Caledonia. Dental status, chewing efficiency, height, weight and waist circumference were clinically recorded at school. The body mass index (BMI) and the waist to height ratio (WtHR) were calculated. For BMI the WHO Cut-offs were used. Twelve years-old participants responded to a questionnaire concerning socio-demographic and behavioural variables. For statistical analysis, the Clinical Oral and Global Health Index (COGHI) was developed and used. Twelve years-old children were categorised into four groups; Oral Diseases (ODG), Obesity (OG), Obesity and Oral Diseases (ODOG) and a Healthy Group (HG). A multivariate analysis was conducted using mixed-effects multinomial logistic regression models.

**Results:**

Prevalence of overweight and obesity was greatly increasing from 6 years (respectively 10.8% [8.8;13.3] and 7.8% [6.0;9.9]) to 12 years (respectively 22.2% [19.9;24.7] and 20.5% [18.2;22.9]) and one third of the 12-yr-olds had an excess of abdominal adiposity. At age 12, 36.6% of the children were healthy (HG), 27.3% had oral diseases (ODG), 19.7% were obese (OG) and 16.5% had both conditions (ODOG). Geographical location, ethnicity, tooth-brushing frequency and masticatory disability were significant risk factors for the OG, ODOG and ODG groups. Ethnicity and masticatory impairment were common risk indicators for the association of oral diseases and obesity.

**Conclusions:**

In NC health promotion programs should be specifically addressed towards Native populations who are particularly exposed to oral diseases and obesity, integrating a multiple risk factors approach, in order to prevent the onset of chronic diseases in adulthood. The impact of masticatory ability on children’s weight status is a major issue for future research.

## Background

Obesity or being overweight and oral diseases such as tooth decay are frequent chronic diseases, not only in disadvantaged groups in developed countries but also in developing countries [1–3]. Both of these conditions are linked to environmental determinants and behavioural risk factors, including some specific dietary habits (intake of sugar-sweetened drinks and frequency of food intake between meals), considered as common for both diseases [4–6]. Besides, it has recently been showed in children that oral diseases (dental caries and gingival inflammation) and tooth loss may cause masticatory dysfunction and thus nutritional problems [7, 8]. Children with missing or painful teeth may limit their food choices because of chewing problems, which results in lower health related quality of life and nutritionally inadequate diets [9]. On one hand, they might not get sufficient nutrition for normal growth leading to undernutrition. On the other hand, the limitation of food choices also may favour excessive intakes of highly processed high fat and high carbohydrate foods contributing to obesity and obesity related diseases. Until now, only a few studies have investigated the relationship between oral diseases and obesity and little interest has been shown in the study of risk factors common to both obesity and oral diseases [10–14].

In French overseas territories such as New Caledonia, this question is of particular interest. Among children and young people, which often consume sugary drinks on a daily basis, oral diseases are frequent [15]. New Caledonia (pop. 268,800) is a French overseas territory with extensive administrative autonomy. The population is a mix of 39% Kanak people (the original inhabitants of New Caledonia), 27% White European people (Caledonians and Metropolitan Frenchmen), 10% Polynesian people (Wallisians essentially), South-East Asian people and people from Vanuatu. There is no available data on childhood obesity in NC, but 67% of adults, especially Polynesians, are overweight [16]. The mean income per inhabitant places New Caledonia within the richest countries of the South Pacific area but there are strong social and economic disparities within the population. There are also health disparities between the various NC ethnic groups, which make up the population, Natives (Kanak and Polynesian) being more affected by oral diseases and rheumatic heart disease, which are correlated with housing conditions [15, 17]. Since 2009, the New Caledonia health authorities and the political decision-makers have been developing health-promoting programs to raise awareness about oral health and to slow down the rise in obesity, particularly among children. This policy aims at preventing the occurrence of metabolic syndrome in teenagers and young adults.

In this context, the first aim of this study was thus to evaluate the prevalence of oral diseases, overweight and obesity in New Caledonian Children (aged 6, 9, 12 years) in order to evaluate the needs for a health promotion program. An ancillary study was further intended to identify individual or environmental risk indicators in NC 12-year-olds that could be common to the groups of children affected by oral diseases or obesity or both diseases. Moreover, it was decided to explore the specific role of masticatory dysfunction on the occurrence of both diseases.

## Methods

This descriptive epidemiological survey evaluated the oral health, stature-weight status and related risk indicators in a national representative sample of 6, 9 and 12-year-old New Caledonian children during the school year 2011–2012.

### Study population

The calculation of the sample size (*n* = 800 for age 6 and 9 children and 1200 for children aged 12) and the sampling method used in this survey (a computerised cluster sampling method with a probability proportional to size) have been described previously [15]. The pool of all the NC elementary and junior high schools was stratified according to the area (South/North/Island) and the type of school (public/private) and 76 schools (50 public and 26 private; 20 in the North region of the main island, 41 in South and 15 in the Islands) were selected. The sample thus included 3138 children (911 6-yr-olds, 923 9-yr-olds and 1304 12-yr-olds). The age group of 12 year olds was chosen to explore the association with risk indicators because it is, according to WHO, the global indicator age group for international comparisons and surveillance of oral disease trends.

### Study variables and data collection

The study was conducted between July 2011 and September 2012 at school. In order to increase the participation rate and ensure the validity of the study, the time for each child’s evaluation (examination and questionnaire) was restricted. The number of questions was limited and simple answering options questions were preferred. Investigators’ calibration and data collection for oral diseases have been described in a previous paper and are summarised below [15]. Seven dentists evaluated the children’s oral status during a clinical examination. Assistants trained for the stature weight data collection accompanied the dentists. Dental caries was diagnosed at the dentinal level (D3) for deciduous and permanent teeth and the number of decayed, missing or filled teeth (DMFT, dft) were calculated [18]. The gingival status was evaluated with the Gingival Index of Loë & Silness and the children were classified in three groups: without gingivitis (score 0 for all sextants), with localised gingivitis (score > 0 in one group of teeth) or with generalised gingivitis (score > 0 for one or both arches) [19]. The presence of mucosal signs of an infectious process was also recorded. Chewing efficiency was evaluated by recording the number of posterior functional dental units (PFU) which has recently shown to impact the masticatory ability in children [7, 9]. The presence of severe oro-facial dysmorphology and the type of breathing (oral/ nasal/ mixed) were also recorded [20]. Prior to the start of the study, all the dentists underwent a two-day training course consisting of projection of pictures illustrating clinical situations and explaining the data collection process. Intra- and inter-examiner agreements were evaluated using Cohen’s Kappa and varied from 0.46 to 0.92 and from 0.69 to 1, depending on the clinical variables. Study variables are detailed in Table 1.

**Table 1:** List of the study variables

	Description of the indicator
Socio-demographic variables	
Age (years)	(Date of examination- date of birth)/365.25
Sex	Female/Male
Region	Islands / North / South
Type of school	Public/Private
Cultural Ethnic group	Melanesian / Polynesian / New Caledonian / Others
Oral diseases (clinical evaluation)	
Gingivitis ^a^	
No	Gingival index = 0 for both arches
Localised	Gingival index > 0 in only one group of teeth
Generalised	Gingival index > 0 for one or both arches
Dental caries ^b^	
No	untreated carious lesion
At least one	untreated carious lesion
Infectious dental process ^c^	
No	infectious dental process
At least one	infectious dental process
Number of posterior functional units (PFU ^d^)	PFU < 4
	4 ≤ PFU < 6
	PFU ≥ 6
Severe oro-facial dysmorphologies ^e^	None
	One or more
Type of breathing	Nasal breathing
	Oral (or mixed) breathing
Stature weight status (clinical evaluation)	
Body mass index BMI	
Under-weight	BMI < -2SD
Normal-weight	-2SD ≤ BMI ≤ 1SD
Over-weight	BMI > 1SD
Obesity	BMI > 2SD
Waist to height ratio WtHr	
Normal	WtHr <0.5
At risk	WtHr ≥0.5
Self-reported oral health and behaviours (questionnaire)	
Frequency of tooth brushing	Less than once a day
	Once or several times a day
Usual drink when thirsty	Water when thirsty
	Sweet drinks when thirsty
Has already smoked	No, has never smoked
	Yes, has already smoked
Lunch at school	No, the child has lunch at home
	Yes, the child has lunch at school
Experience with dental care	No, has never visited a dentist
	Yes, has already visited a dentist
Dental anxiety (VAS 0 to 10)	No or low anxiety (score ≤ 3)
	Yes moderate to high anxiety (score > 3)
Experience of recent dental pain	No
	Yes
Prevention at school	No experience
	Already experienced
Feel able to eat all kind of foods	No, problems in eating some kind of foods
	Yes, able to eat all kind of food

^a^ Løe and Silness index [19]

^b^ Stage 3 or 4 of Ekstrand classification [18]

^c^ Presence of an abscess, a cellulite, a tooth with pulpal exposure or a fistula

^d^ Number of Pairs of posterior teeth with at least one contact area during chewing [7, 9]

^e^ > 2 years from the normal age of eruption or dental overcrowding >4 mm from the occlusion plane, > 1 mm negative over-jet, > 6 mm positive over-jet, Unilateral or bilateral cross-bite and complete overbite, > 6 mm open overbite [20]

For measuring the stature weight status, assistants used tape measures and SECA® digital scales. Children wore light clothing but no shoes. The body mass index (BMI in Kg/m^2^) and the waist to height ratio (WtHR) were calculated. For each child, weight and height were collected and BMI-for-age Z-scores were calculated based on the updated WHO reference [21, 22]. Children with BMI Z-score between ≥ − 2 and ≤ + 1 were classified as normal weight; those with Z-score between > +1 and ≤ +2 were considered overweight; and those with Z-score > +2 as obese. The waist-height ratio (WHtR) was calculated by dividing the waist circumference by the height. A WtHR value of ≥0.5 was considered as a risk factor for metabolic syndrome [22, 23]. In order to allow comparisons with other epidemiological studies, the prevalence of overweight and obesity were also calculated using the international obesity task force (IOTF) definition [24].

Socio-demographic variables (gender, date of birth, area of residence, public or private school) were obtained from the school register. Moreover, children responded to a self-administered questionnaire on their behaviours (lunch at school/at home, frequency of tooth brushing, usual drink when thirsty, has already smoked/not), experience with dental care (already visited a dentist, level of dental anxiety, prevention made at school/not) and reported oral condition (recent experience of dental pain, self-perception of chewing ability). Finally, children were asked to which main cultural/ ethnic group (Kanak / Polynesian / Caledonian / European, Asian / Other) they felt they belonged to. In NC, ethnic characteristics are prominent compared with social and economic ones and ethnic statistics have been authorised, not the case in any other French territories.

## Data analysis

Data entry was duplicated and errors were corrected. The statistical analyses were performed using Stata software (version 13, StataCorp, College Station TX, USA). Prevalence of oral diseases, abdominal and general over-weight and obesity were calculated with 95% confidence intervals (CI) to describe the Caledonian children’s health status. Mean DMFT, BMI and WtHr were calculated for all the children for whom all data were complete.

Then, for the 12-year-olds, indicators of oral and stature/weight status were associated to produce a new combined index which was built for the needs of the study: The Clinical Oral and Global Health Index (COGHI). An algorithm was applied using logical association (If/then/else) to categorise the children into four groups: the group with Oral Diseases (ODG), the Obesity group (OG, including obese children), the Obesity and Oral Diseases group (ODOG) and a Healthy control group (HG). Construction of the COGHI subgroups is detailed Table 2. A sensitivity analysis showed that excluding the underweight children from the COGHI groups, had no impact on the bivariate and multivariate analyses. Thus, children considered as underweight and children for whom all the health indicators were not available were excluded of the algorithm.

**Table 2:** Definition of the “Clinical Oral and Global Health Index” groups and proportion of 12-yr-olds children in each group

COGHI Groups	Criteria		Proportion of children (*N* = 1165)
Healthy group (HG)			36.6%
No oral diseases Not obese		No untreated dental caries (D3T = 0)	
	and	No gingivitis (localised or generalised)	
	and	No mucosal sign of infection	
	and	Not obese (BMI ≤ 2 SD)	
	and	No risk for metabolic syndrome (WtHr < 0.5)	
Oral Diseases group (ODG)			27.3%
Oral diseases Not obese		At least one untreated carious lesion (D3T > 0)	
	or	Generalised gingivitis (Gingival index > 0 for one or both arches)	
	or	Mucosal sign(s) of infection	
	and	Not obese (BMI ≤ 2 SD)	
	and	No risk for metabolic syndrome (WtHr < 0.5)	
Obesity group (OG)			19.7%
Obese No oral diseases		Obesity (BMI > 2 SD)	
	or	Risk for metabolic syndrome (WtHr ≥ 0.5)	
	and	No untreated dental caries (D3T = 0)	
	and	No gingivitis (localised or generalised)	
	and	No mucosal sign of infection	
Oral Diseases/Obesity group (ODOG)			16.5%
Oral diseases Obese		At least one untreated carious lesion (D3T > 0)	
	or	Generalised gingivitis (Gingival index > 0 for one or both arches)	
	or	Mucosal sign(s) of infection	
	and	Obesity (BMI > 2 SD)	
	or	Risk for metabolic syndrome (WtHr ≥ 0.5)	

The associations between oral diseases or obesity and explanatory independent variables were analysed using random-effects models integrating school and examiner parameters as random effects [25]. Then, a multivariate analysis was conducted to test comparisons between the three disease groups and the healthy comparison group. Mixed-effects multinomial ordinal logistic regression models were performed to identify common indicators associated with the groups. The GLLAMM (Generalized Linear Latent And Mixed Models) package implemented in Stata Software was used [26]. These analyses were applied based on factors considered significant in bivariate analysis and according to clinical relevance. In an attempt to reduce the effect of potential bias, a propensity score was performed in order to balance all relevant variables related to masticatory ability. The probability of having a “number of functional units” (<6) was calculated including covariates known to be clinically relevant (mean DMFT, presence of infectious process, presence of severe oro-facial dysmorphology, perception of recent dental pain, presence of mouth breathing, perception of chewing difficulties, presence of dental anxiety, number of permanent teeth). The propensity score estimation was considered as covariate in the multivariate analysis described previously. The results are reported using the estimated coefficients transformed into relative-risk ratios (RRR), with 95% CI and *P*-values (p). The level of statistical significance (type I error α) was set at 0.05.

The WHO definitions were used to define obese children in the statistical analyses.

## Results

### Prevalence of oral diseases and being overweight or obese

Of the 3138 children initially selected, 2734 were examined (744 6-yr-olds, 789 9-yr-olds and 1201 12-yr-olds). The Inter-observer coefficient (ICC) was 9.3% (2.1–32.6%) for the whole group of examiners. The ICC was lowered to 4.1% (0.8%–18%) when one observer who examined 32 children in the Island area was deleted. Prevalence of oral diseases, overweight and obesity, according to WHO cut-offs, for 6, 9 and 12-year-old children are given Table 3 and mean DMFT, BMI and WtHr are presented Table 4. Prevalence of oral diseases, except gingivitis, seemed to increase between 6 and 9-year-olds, and to decrease between 9 and 12-year-olds. Among the 12 years-old group, 47% of the children had at least one untreated carious lesion, 16% suffered from mucosal infectious lesions and 62% showed signs of localised or extended gingivitis. Even if oral breathing or masticatory impairment tended to decrease between age 9 and 12, severe orofacial dysmorphologies were present in 31% of the 12-yr-olds. Ten percent continued to breathe through the mouth and almost 20% had less than 6 posterior functional dental units to chew (including 2.7% with less than 4 PFU). The prevalence of overweight (including obesity) increased greatly between 6 and 9 years and obesity particularly between 9 and 12 years. The proportion of under-weight children was stable at all ages (1.6% to 1.8%). The prevalence of overweight and obesity was higher when measured with the IOTF classification (overweight: 25.5 [23.0–28.1] and obesity: 25.5 [23.0–28.1]) instead of the WHO cut-offs in the 12-yr-olds. Approximately one third of the 12-yr-olds suffered from an excess of abdominal adiposity associated with overweight or obesity. At age 12, mean BMI was 20.9 (4.9) (girls: 21.3 (5.0) and boys: 20.6 (4.7)) and mean WtHr was 0.49 (0.07) for the whole sample (including normal-weight and under-weight children). The mean WtHr was 0.50 (0.04) for over-weight children and 0.58 (0.06) for obese children. Respectively 54% and 96% of the overweight and obese 12 years old children had a WtHr higher than 0.5.

**Table 3:** Prevalence of oral disease,s overweight and obesity at 6, 9 and 12 years

	Age 6 yr		Age 9 yr		Age 12 yr	
	*N*	*% [95% CI]*	*N*	*% [95% CI]*	*N*	*% [95% CI]*
At least one untreated carious lesion	744	57.7 [54.0;61.2]	789	59.9 [56.4;63.4]	1201	46.9 [44.0;49.7]
Localised gingivitis	731	44.6 [41.0;48.3]	787	43.6 [40.1;47.1]	1201	36.8 [34.1;39.6]
Generalised gingivitis		8.5 [6.6;10.7]		16.0 [13.5;18.8]		25.4 [22.9;27.9]
At least one infectious dental process	737	13.8 [11.4;16.5]	782	12.7 [10.4;15.2]	1198	16.3 [14.3;18.5]
At least one severe oro-facial dysmorphology	739	31.1[27.8;34.6]	782	38.6 [35.2;42.1]	1198	31.5 [29.0;34.2]
Oral (or mixed) breathing	717	28.5 [25.2;31.9]	770	27.8 [24.7;31.1]	1193	10.6 [8.9;12.5]
Number of posterior functional units	NI		754		1201	
PFU < 4				15.1 [12.6;17.9]		2.7 [1.9;3.8]
4 ≤ PFU < 6				43.1 [39.5;46.7]		17.0 [14.9;19.2]
PFU ≥ 6				41.8 [38.2;45.4]		80.3 [77.9;82.5]
According to WHO cut-offs	721		773		1182	
Under-weight		1.8 [1.1;3.1]		1.6 [0.9;2.7]		1.8 [1.1;2.7]
Normal-weight		79.6 [76.5;82.4]		68.9 [65.6;72.1]		55.5 [52.6;58.3]
Over-weight		10.8 [8.8;13.3]		18.1 [15.6;21.0]		22.2 [19.9–24.7]
Obesity		7.8 [6.0;9.9]		11.4 [9.3;13.8]		20.5 [18.2–22.9]
WtHr ≥ 0.5	735	29.4 [26.2;32.8]	756	29.0 [25.8;32.3]	1200	34.3 [31.6–37.1]

*N* number, *%* proportion, *NI* not investigated, *CI* confidence interval

**Table 4:** Stature-Weight status and caries experience of children aged 6, 9 and 12 years

	Age 6 yr		Age 9 yr		Age 12 yr	
	*N*	*mean[CI]*	*N*	*mean[CI]*	*N*	*mean[CI]*
DMFT	*594*	0.09 [0.05;0.12]	*789*	0.76 [0.67;0.85]	*1201*	2.09 [1.93;2.25]
BMI	*740*	16.49 [16.33;16.65]	*789*	18.39 [18.14;18.64]	*1182*	20.9 [20.6;21.2]
WtHr	*735*	0.490 [0.487;0.493]	*756*	0.486 [0.482;0.490]	*1198*	0.49 [0.48;0.49]

BMI and WtHr Means are given for the whole sample (under-, normal-, over-weight and obese children)

*N* number, *NI* not investigated

Among the 1165 children for whom data were complete, 36.6% of the children were healthy (HG), 27.3% had oral diseases but were not obese (ODG), 19.7% were obese but had a good oral status (OG) and 16.5% were affected with both chronic conditions (ODOG).

### Common risk indicators for oral disease and obesity

The bivariate analysis showed that environment was significantly associated with the children’s health status. Children from the Islands were highly represented in the ODG group and children from the North in the ODG and ODOG groups. The ethnic groups were significant determinants for the ODG and ODOG groups, Polynesian children being particularly at risk for obesity (Table 5). Behavioural variables, such as tooth brushing less than once a day or smoking, were significant explanatory variables for the groups of children with oral diseases (ODG, ODOG). Inversely, the variable that explored the children nutritional habits (usual sweet drinks consumption or lunch setting) was not a significant risk indicator for the obesity (OG) group. Variables related to experience with dental care or prevention were not related to the ODG and ODOG groups.

**Table 5:** Association between risk indicators and prevalence of oral diseases and obesity (bivariate analysis)

Groups of the Clinical Oral and General Health Index (COGHI)	Healthy ^R^	Oral diseases	Obesity	Oral diseases & Obesity	Total N (%)
Sex (*n* = 1155)					
Male^R^	35.9%	28.7%	18.9%	16.3%	566 (49.0)
Female	37.2%	25.8%	20.4%	16.6%	589 (51.0)
Geographical area (*n* = 1130)					
Islands	33.0%	39.6% **	15.1%	12.3%	71 (6.3)
North	27.2%	42.7%***	11.8%	18.3%*	246 (21.8)
South ^R^	39.8%	21.03%	22.6%	16.5%	813 (71.9)
Cultural ethnic group (*n* = 1128)					
Melanesian	38.1%	26.3%*	22.1%	13.5%**	312 (27.7)
Caledonian	34.8%	32.1%**	16.0%	17.0%***	505 (44.8)
Polynesian	27.5%	19.8%*	25.1%**	27.5%***	207 (18.3)
Others^R^	56.7%	20.2%	20.2%	2.9%	104 (9.2)
Type of school (*n* = 1166)					
Public ^R^	37.2%	26.1%	20.8%	15.9%	881 (75.6)
Private	34.5%	31.0%	16.2%	18.3%	285 (24.4)
Tooth-brushing frequency (*n* = 1137)					
≥1/day^R^	41.7%	23.0%	21.5%	13.8%	631(55.5)
< 1/day	29.3%	32.4%***	18.0%	20.4%***	506 (44.5)
Use of sweet drinks (*n* = 1163)					
Yes	35.1%	31.0%	16.9%	16.9%	413 (35.5)
No^R^	37.3%	25.3%	21.1%	16.3%	750 (64.5)
Lunch at school (*n* = 1154)					
Yes^R^	37.1%	25.6%	20.2%	17.1%	1038 (89.9)
No	32.8%	41.4%**	12.9%	12.9%	116 (10.1)
Smoking habits (*n* = 1106)					
Already smoked	25.0%	35.6%**	15.9%	23.5%**	132 (11.9)
Never^R^	37.7%	26.7%	19.9%	15.7%	974 (88.1)
Dental attendance (*n* = 1153)					
Already visited a dentist	37.1%	26.9%	18.9%	17.1%	957 (83.0)
Never^R^	35.2%	29.1%	21.9%	13.8%	196 (17.0)
Dental anxiety (*n* = 1165)					
Anxiety	36.0%	28.0%	18.6%	17.4%	414 (35.5)
No anxiety (score ≤ 3)^R^	36.9%	26.9%	20.2%	16.0%	751(64.5)
Prevention at school (*n* = 1002)					
No experience	36.9%	26.2%	20.0%	16.9%	691 (68.9)
Already experienced^R^	34.7%	30.5%	20.3%	14.5%	311 (31.1)

The groups Oral disease, Obesity, Oral disease & Obesity are compared with the Healthy group

The bivariate analyses integrate the school and investigator effects

For the explanatory variables, each category is compared with a reference category indicated with a R

Proportions (%) are calculated per line for the different COGHI groups and per column for the total

***P < 10^–^^4^, **P < 0.01, *P ≤ 0.05

The propensity score evaluated the probability of the children having a low number of posterior functional units (<6 PFU) (Table 6). Among the covariates integrated in the model, the presence of mucosal signs of an infectious process, oral breathing, dental anxiety, self-perceived chewing difficulties significantly influenced the score. The number of dental functional units (<6 versus ≥ 6) was also related to the number of present permanent teeth (23.4 vs 26.3, *p* < 0,001) but not to the number of carious, filled or missing teeth (DMFT), nor to the presence of severe orofacial dysmorphology.

**Table 6:** Propensity score, probability of having a low number of posterior functional units (<6)

	RRR	*P* value	[95% Conf. Interval]
Mean DMFT	1.01	0.74	0.94–1.08
Presence of infectious dental process	**1.93**	**0.01**	1.14–3.27
Perception of recent dental pain	0.84	0.43	0.55–1.29
Presence of oro-facial dysmorphology	1.07	0.72	0.73–1.57
Presence of mouth breathing	**2.17**	**0.00** **4**	1.28–3.67
Perception of chewing difficulties	**1.76**	**0.003**	1.21–2.56
Dental anxiety	**1.74**	**0.04**	1.19–2.53
Number of permanent teeth	**0.74**	**0.001**	0.71–0.78

Logistic regression

Significant RRR and corresponding *p* values (<0.05) are captured in bold

The multi-dimensional analysis showed that cultural attachment, the region of residence and oral hygiene habits were significantly associated with the health status of children (Table 7). Indeed, comparison between ODOG and HG showed that Natives (Caledonians, Melanesians, Polynesians) were more exposed to both oral diseases and being obese than non-Natives. Ethnicity was the only significant variable related to the OG group and Polynesian children were particularly affected by obesity. Concerning the ODG group, it appeared that children from the North and the Islands regions had respectively a three-times and two-times higher risk of being affected with oral diseases than children in the South. Tooth brushing frequency was a common risk factor for children with oral diseases (ODG) and for children with oral diseases and obesity (ODOG). The propensity score which evaluated the masticatory ability of the children was a significant risk indicator for the OG and the ODOG groups.

**Table 7:** Multivariate analysis: Risk indicators associated with oral diseases or being obese

Groups of the Clinical Oral and General Health Index (COGHI)	Oral disease	Obesity	Oral disease & Obesity
Sex: girl vs boy	0.81 (0.55–1.21)	0.80 (0.52–1.22)	0.82 (0.52–1.29)
Geographical area:			
North vs South Islands vs South	3.27 (1.98–5.40)*** 2.20 (1.09–4.43)*	0.78 (0.41–1.47) 0.61 (0.24–1.54)	1.87 (1.04–3.38)* 1.39 (0.59–3.28)
Cultural ethnic group			
Melanesian vs others	1.23 (0.59–2.59)	1.91 (0.89–4.09) *p* = 0.09	5.19 (1.15–23.50) *
Caledonian vs others	1.58 (0.79–3.17)	1.63 (0.78–3.39)	9.20 (2.11–40.08) **
Polynesian vs others	1.90 (0.84–4.30)	2.48 (1.11–5.55) *	16.81 (3.71–76.20)***
Type of school	0.79 (0.50–1.27)	0.79 (0.46–1.35)	0.99 (0.59–1.68)
Private vs Public			
Toothbrushing frequency	1.89 (1.26–2.83)**	1.09 (0.70–1.69)	1.75 (1.10–2.78)*
<1/day vs ≥1/day			
Smoking habits			
Already smoked vs Never	1.41 (0.76–2.63)	0.95 (0.45–2.01)	1.72 (0.88–3.37)
Use of sweet drinks			
Yes vs No	1.11 (0.66–1.87)	0.77 (0.49–1.21)	1.10 (0.70–1.74)
Dental attendance	1.07 (0 .63–1.83)	0.75 (0.43–1.32)	1.59 (0.83–3.04)
Already visited a dentist vs never			
Prevention at school	0.85 (0.56–1.27)	0.96 (0.62–1.50)	1.20 (0.74–1.94)
No vs Already experienced			
Propensity score for FU < 6	2.14 (0.70–6.44)	8.49 (2.11–34.15)**	7.01 (1.49–33.00)*
Dental anxiety Anxiety vs No anxiety (≤3)	1.05 (0.69–1.59)	0.82 (0.51–1.29)	1.24 (0.78–1.99)
Lunch at school	0.95 (0.50–1.83)	0.93 (0.39–2.26)	0.61 (0.26–1.43)
No vs Yes			

Mixed-effects multinomial ordinal logistic regression models

The groups Oral disease, Obesity, Oral disease & Obesity are compared with the Healthy group

The school and investigator effects are taken into account for each comparison

***P < 10–4, **P < 0.01, *P ≤ 0.05

## Discussion

This study provides useful epidemiological data about obesity in New Caledonian children aged 6 to 12 years. A high prevalence was observed with 8% to 20% of the children being obese and the prevalence was increasing with the age of the children. At age 6 and 9, the prevalence of overweight and obesity, according to WHO cut-offs were similar to the most impacted European countries in 2013 [27]. At age 12, the prevalence of overweight (excluding obesity) (22.2%) was higher than that observed in metropolitan France in 2009 (19.7% at age 12 years) [28] but similar to the average worldwide values calculated for children under 20 in developed countries in 2013 (22.6% to 23.8%,) [2]. In this study, BMI and WtHr data were associated to evaluate accurately the risk of metabolic syndrome. In New Caledonia, one third of the children cumulated excess abdominal adiposity (WtHr >0.5) and general overweight, which are associated with an increased risk for metabolic syndrome, diabetes and cardiovascular comorbidities [23]. Results also indicated that oral diseases remained frequent in NC children. Caries experience at 6, 9 and 12 years was similar to that of other developed countries such as the USA or Europe [29, 30].

Approximately one child on six was thus affected with oral diseases and obesity at 12 years. Obesity or being overweight is key to identifying children with higher metabolic and vascular risk. Nevertheless, only a few studies have examined the association of metabolic syndrome with periodontal diseases (including its first stage, gingival inflammation) and dental caries. Metabolic syndrome seems to be associated with the presence of periodontal pockets, decayed teeth and to the incidence of tooth loss among middle-aged adults [31, 32]. A study has found correlations between the risk of metabolic syndrome and high salivary S. Mutans counts in children [33]. Childhood obesity was also pointed to be associated with reduced flow rate of stimulated saliva and thus being a potential risk factor for dental caries [34].

Obesity in childhood is associated with immediate adverse health and psychosocial outcomes. Obesity can affect children’s educational attainment and quality of life [35]. Obesity has also long-terms negative health, social and even economic consequences. Obese children are likely to remain obese as adults and are thus at risk of chronic illnesses like diabetes, coronary heart diseases or some types of cancer [36]. The life course approach to ageing also suggests that health status at one time is not only dependent on contemporary environmental or behavioural determinants but also on the level of health in earlier life, which depends on developmental processes and early environmental influences [37]. It is thus important in NC to prevent children being overweight but also to treat children who are already obese, for their own well-being, their future health but also that of their children.

Oral diseases also are associated with adverse health and psychosocial outcomes. Children with poor oral health are more likely to experience dental pain and sepsis, to miss school and to perform poorly in school [38]. Adult oral health is associated with intergenerational factors and various aspects of people’s beliefs, social status, dental attendance and self-care operating (as tooth brushing habits) from childhood [39]. In this field, the impact of oral health on general health should not be underestimated. Being edentulous was identified in 2013 as one of the top 25 causes of an increased number of “years lived with disability” [1]. Our results thus indicate that it is important to integrate oral health into the health promotion programs that will need to be conducted in NC to prevent and treat health problems related to obesity.

One of the aims of the study was to identify high-risk population groups through environmental determinants (cultural belonging, area of residence, type of school, prevention at school, lunch setting) in order to target appropriately future health promotion measures. Geographical residence and ethnicity were significant explanatory determinants for both diseases. Native children were at higher risk of suffering from obesity and oral diseases, Polynesian children were particularly at risk for obesity, like it has been described by a previous study conducted among Pacific Islands Natives [40]. The role of ethnicity in the observed health disparities may be related to various genetic, cultural or social determinants. The way of life and health status of Polynesian parents during pregnancy or during infancy may have influenced the development of obesity and oral diseases observed in pre-teens [41]. The identification of cultural enablers and barriers to the implementation of health promotion interventions for these high-risk Native populations are of particular importance in this context [42]. Moreover it would be helpful to quantify the impact of recent and sometimes abrupt lifestyle changes for Native people of the Pacific islands, from their traditional nutritious habits and daily activity to the modern societal organization.

The only study nutritional variable (usual consumption of sugary drinks) was not found to be a significant risk indicator for oral diseases and/or obesity. These results are not in accordance with other findings from large observational studies that supported a link between consumption of sugar-sweetened beverages and obesity [14]. Moreover, even if the relationship is complex, frequent sugar consumption is considered to be an important etiological factor for dental caries [28]. Nutritional habits such as meal skipping and consumption of sugary drinks, milk or fresh vegetables have also been shown to be significantly correlated with caries experience or gingivitis while taking into account weight status [43].

Interactions between dental status and nature of food have dominant role as an entraining stimulus for metabolic rhythms, the timing of daily food intake and the fidelity of food entrainment mechanisms. During food oral processing, the teeth are not simple tools that mechanically reduce the food to particles and mix saliva and the food to produce an easy-to-swallow bolus. They are also essential to the neuromotor control of chewing and swallowing, through the periodontal and pulpal sensory receptors that are triggered during interarch contacts. The number of PFU is related to the number of interach contacts. In nutrition studies, chewing difficulties are rarely considered as explaining factors for food selection and feeding behavior. In this study, masticatory ability was integrated in the multivariate analysis as an explanatory variable by using a Propensity score which evaluated the « probability of having a low number of functional units ». Masticatory disability was found to be a significant indicator for the presence of obesity and the association of oral diseases and obesity. Children at age 12 are characterised by recent changes in the posterior dentition leading to a reduced number of teeth in occlusion, which interact with masticatory ability. Our results show that infectious processes of dental origin and the persistence of mouth breathing also reduced chewing ability, which may secondarily cause avoidance of hard and fibrous foods like fruits or vegetables [44]. The limitation of food choices may then favour excessive intakes of highly processed high fat and high carbohydrate foods contributing to obesity [9].

The prevalence of children having already smoked (12%) in NC was slightly higher than that observed in a sample of 11–13 years old children in the city of Marseille (France) where 10.5% declared having already smoked [45]. Smoking may have a negative impact on adolescents’ oral health although the level of evidence is poor [46]. In addition to passive exposure from maternal smoking, stress-related behaviours like smoking represent risk factors for obesity among adolescents or young adults [47]. The future NC Health promotion programme should integrate a multiple risk factors approach with a special interest on tobacco use as well as it should target the most affected populations.

The study design had a number of limitations. The findings were based on self-reported data from the children, mainly dichotomised, which may have limited the precision of the information collected, particularly for nutritional variables. It is also likely that the true frequency of some behaviours, like tobacco-use, was under-reported due to the children’s reluctance to admit “bad habits” in front of health or educational professionals. Social status was not investigated because the children’s answers would not have been reliable enough and the parents’ response rate was highly uncertain. It should also be noted that it is difficult to make comparisons about the prevalence of obesity due to variability in the classifications used. The cut-off values used by the International Obesity Task Force (IOTF) give different prevalence values than for the WHO cut points [24].

## Conclusions

This survey showed that the prevalence of both overweight and obesity were high in New Caledonian children, indicating the need to implement a comprehensive health promotion programme. Health promoting interventions are usually based on the common risk factor approach, which aims at improving health by preventing the onset or the worsening of several chronic diseases. Before implementing a pertinent health promotion policy in NC, it was thus necessary to target the most affected population groups and to identify the common risk indicators for children suffering from cumulative diseases. In this study, ethnicity was identified to impact deeply the distribution of oral diseases and obesity in New Caledonian 12-year-olds.

The Pacific islands’ original populations (Kanak and Polynesian) were more likely to be affected with one or both chronic diseases. Those ethnic groups were already known to be more affected with other metabolic and infectious diseases in adulthood. However, ethnic health disparities are still not integrated in NC health policies and strategies. To help understand the mechanism of health disparities in NC, the cumulative or independent effect of social and ethnic gradient should be clarified.

Based on these results, local stakeholders have now developed a health promotion program aimed at preventing metabolic syndrome, based on inter-sectorial collaboration and that integrates oral health issues. The promotion of a healthy nutrition is a key issue as the consumption of free sugars is high in NC, particularly the use of soft drinks. Nevertheless, methodological limits of this ancillary descriptive study did not allow to explore thoroughly this aspect. Moreover, the children’s masticatory ability is obviously a major issue for future research.

## Abbreviations

BMI: Body mass index
CI: Confidence interval
COGHI: Clinical oral and global health index
D3: Dentinal threshold level
Dft: Number of decayed or filled temporary teeth
DMFT: Number of missing, decayed or filled permanent teeth
HG: Healthy group according to COGHI
IOTF: International task force
NC: New Caledonia
ODG: Oral disease group according to COGHI
ODOG: Oral disease and obesity group according to COGHI
OG: Obesity group according to COGHI
PFU: Number of posterior functional dental units
RRR: Relative risk ratio
SD: Standard deviation
WHO: World health organization
WtHR: Waist to height ratio
